# Safe and competent nursing care: An argument for a minimum standard?

**DOI:** 10.1177/0969733020919137

**Published:** 2020-05-18

**Authors:** Siri Tønnessen, Anne Scott, Per Nortvedt

**Affiliations:** 7928 University of South-Eastern Norway, Norway; 8799 National University of Ireland Galway, Ireland; University of Oslo, Norway

**Keywords:** Fundamental nursing care, human rights to nursing care, minimum standards of nursing care, missed care, rationing, safe and competent nursing care, values in nursing care

## Abstract

There is no agreed minimum standard with regard to what is considered safe, competent nursing care. Limited resources and organizational constraints make it challenging to develop a minimum standard. As part of their everyday practice, nurses have to ration nursing care and prioritize what care to postpone, leave out, and/or omit. In developed countries where public healthcare is tax-funded, a minimum level of healthcare is a patient right; however, what this entails in a given patient’s actual situation is unclear. Thus, both patients and nurses would benefit from the development of a minimum standard of nursing care. Clarity on this matter is also of ethical and legal concern. In this article, we explore the case for developing a minimum standard to ensure safe and competent nursing care services. Any such standard must encompass knowledge of basic principles of clinical nursing and preservation of moral values, as well as managerial issues, such as manpower planning, skill-mix, and time to care. In order for such standards to aid in providing safe and competent nursing care, they should be in compliance with accepted evidence-based nursing knowledge, based on patients’ needs and legal rights to healthcare and on nurses’ codes of ethics. That is, a minimum standard must uphold a satisfactory level of quality in terms of both professionalism and ethics. Rather than being fixed, the minimum standard should be adjusted according to patients’ needs in different settings and may thus be different in different contexts and countries.

## Introduction

Today, nurses in all kinds of positions throughout healthcare organizations experience the impact of fiscal constraints, including pressure on staffing. Healthcare resources are limited, and nursing care is consequently a limited resource.^1,2^ Allocation of limited resources generally implies a difficult process of prioritization and/or rationing, at all levels from the macro national allocation through the regional, service, and meso-allocation level to the micro-unit/bedside levels.^3^ Prioritization and rationing on a micro level (bedside) often involves choosing which patients to prioritize and which elements of nursing care to omit, delay, or provide to the patient.^4^ Such decisions, particularly decisions to omit or delay care, can cause significant moral distress to the nurses involved.^5^

Schubert et al.^6^ define rationing of nursing care as “the failure to carry out necessary nursing tasks due to inadequate time, staffing level and/or skill mix.” There is also growing evidence that the elements of nursing care most frequently left undone or missed are the so-called “basic” and “softer” elements of care, such as patient hygiene, comfort care, patient education, and discharge planning.^7,8^ These are elements of care that make the patient feel better, feel cared for. These elements of care lead to patients perceiving that ‘they are in good hands’—that is, that they are being cared for by competent, caring staff.^9^ It can be argued that care that helps the patient feel better, feel cared for, is care that is rooted in ethically sound practice—recognizing what is good for the patient from a care perspective.^10^

It is well acknowledged and understood that the clinical and ethical dimensions of nursing care are intimately intertwined, and this is because effective, competent (skilled care that is safe for recipients), humane nursing care is not only good (and therefore the desired) clinical nursing practice it is also by definition ethically sound nursing practice. Our ability to separate the clinical and ethical dimensions from each other is at best very difficult and some would argue neither possible nor desirable.^11,12^ Nonetheless what one would consider “necessary nursing care,” the nursing care required by a patient to feel safe and cared for remains unclear. This lack of clarity is perhaps due to a number of issues such as the variation in individual patient need, the ability of particular health systems to meet the variety of patient needs effectively, and/or the willingness of national governments to fund the types and levels of care required to meet not only patient/client need but also patient/client expectation. As far as we know, an articulated, agreed upon minimum standard of nursing care, in other words an agreed upon description of “necessary nursing care” to ensure safe and competent nursing care, does not exist—though descriptions of what nursing is clearly do exist.^13–15^ In developed countries with tax-funded public healthcare systems, however, patients have a right to a minimum standard of healthcare, including nursing care.^16^ Thus, it seems appropriate to discuss what this might look like from a nursing perspective—that is, what types of nursing care services are necessary to be able to ensure a minimum standard of safe, competent nursing care?

In this article, we wish to explore the value in articulating a minimum standard of safe, competent nursing care and suggest what such a standard might encompass. We use Norwegian healthcare legislation to illustrate how safe, competent nursing care can be anchored in an actual jurisdiction and how this complies with relevant professional and ethical principles governing nursing. In the Norwegian context, this includes the relevant health legislation (acts and regulations) and governmental documents (official governmental reports and white papers) as well as the requirements of the Norwegian Nurses Organization. We explore both the content of a minimum standard of safe, competent nursing care and the values underpinning such a standard. Moreover, we argue that access to a minimum standard of safe, competent nursing care is in accordance with the human right to healthcare and must coincide with professional codes of nursing ethics and the requirement of reliable and caring services (as discussed in the following). Finally, we emphasize that an international minimum standard cannot be a fixed quantitative or qualitative standard. It must have an inherent flexibility and be subject to discussion so that it can be adjusted to various clinical settings and sociopolitical contexts. This is important in order to meet individual patients’ needs, recognizing the reality of different patients and resourcing contexts and consequent differing patient expectations and political capacities to meet those expectations. First, however, we outline the matter of concern on the individual level, that is, for patients, nurses and, more briefly, for next of kin. We then move on to consider the issues for nursing in general, as well as how such matters relate to legislation and the human right to healthcare.

It seems timely to address this matter in a context where increasing attention is being paid to the reality of missed nursing care/nursing care left undone/covert rationing of nursing care^1^ and the consequent implications for both patient experience of healthcare and for patient safety.^1,6,17^

## Matters of concern

There is a gap between people’s need for healthcare services and the resources allocated to ensure patients receive safe, competent nursing care.^1,18^ When resources are scarce, staffing levels are often low, leading to time constraints for nurses providing nursing care.^19^ Limited time to meet patients’ needs consequently implies that nurses must prioritize and ration nursing services. There are many definitions of what it means to prioritize and ration nursing and healthcare services.^3,4,20,21^ For the purpose of this article, which is to suggest an approach to determining what constitutes safe, competent nursing care, whether we call the process rationing or prioritization is not the key issue. What is at stake, rather, is that due to rationing and prioritization, patients potentially do not receive safe, competent nursing care. Many studies indicate that patients’ needs for nursing care are not met because nursing care and tasks are missed, left undone or unfinished, or care is delayed.^1,3,6,22–24^ When nursing care is rationed, missed or left undone, or unfinished, this has negative consequences for patients, nurses, and next of kin. Therefore, there needs to be a discussion regarding what can be considered safe, competent nursing care and how we might think about the idea of a human right to safe competent care.

### Matter of concern for patients

Research shows how rationing and missed nursing care have serious consequences for patients. Studies from hospital settings indicate associations between rationing and/or missed nursing care and patient safety, illuminating a variety of negative patient outcomes including higher risk-adjusted in-hospital mortality.^1,6,17^ Studies also indicate adverse event rates related to medication errors, patient falls, pressure ulcers, and nosocomial infections.^7,25,26^ Implications for patients also relate to access to care and unfair or inconsistent decisions on patient care.^27^ Furthermore, as indicated above, research across European hospital settings elucidates how psychosocial needs, planning, and documenting care are tasks most commonly left undone,^7^ thus indicating failure to meet patients’ psychosocial needs, deficits in discharge planning, and difficulties in follow-up. When quality of care is compromised, it may lead to long-term morbidity.^28,29^

In community settings, studies have shown how dying patients have not been properly attended to and left to die alone. Their need for nursing care such as attending to bed sores, performing prophylactic measures, and providing nutrition, spiritual care, communication, and information is not met.^30,31^ In homecare, patients as well as healthcare professionals describe psychosocial needs not being met and services being delayed, denied, diluted, and delegated to substitute staff with less training.^32^ This leads to unpredictable service provision with lowered quality of care. Such care threatens patients’ autonomy and dignity and raises questions regarding the adequacy and sufficiency of the service.^33–35^ Given the potentially severe consequences for patients of the rationing of nursing care, it is crucial to discuss the threshold for what might be considered safe, competent nursing care to enable nurses to provide adequate nursing services, thus ensuring patient safety and preventing adverse events.

### Matter of concern for nurses

For nurses, not being able to provide care due to limited resources is a situation that entails difficult decision-making processes.^36^ Studies on nurses’ rationing report that nurses experience moral frustration and distress when facing the value conflict of having to choose between patients, including choices regarding which fundamental nursing care elements will be left undone, such as psychosocial and spiritual care, nutrition, or oral hygiene.^34^ These value conflicts result from complex situations where nurses have to take into account several aspects related to fundamental nursing care, such as consequences, benefits, urgency, effectiveness, and harm, to name a few. Facing such a conflict implies choosing between alternatives, none of which are particularly satisfying:^36^ for example, when district nurses have to choose between the older woman who needs help with her diabetes medication or the frail older man who has dementia and needs help to make it to day care in time. Which one does the nurse prioritize and why? Hence, rationing and prioritization affect many parties,^32^ usually result in less care/poorer care for one or more persons, and, in the worst cases, have serious consequences for those involved. Furthermore, because time is limited, these decisions must be made on the spot, without time to consider and reflect.^20^

Research indicates that higher levels of omitted nursing care are associated with adverse outcomes for nurses, including reduced job satisfaction and increased turnover.^7,17^ Rationing might imply compromising patients’ right to healthcare, which conflicts with personal and professional values.^37^ Not being able to provide care in compliance with professional and personal values challenges nurses’ ethical and moral value systems, ultimately resulting in role conflict, guilt, ethical dilemmas, moral strain, and moral distress.^38^ Hence, missed care due to rationing has an impact on professional practice^39^ and increases the experience of moral distress.^40^

As can be expected, nurses have voiced their need for support in these difficult situations.^41^ Various forms of clinical ethics support such as individual ethics consultants, ethics committees, moral case deliberation, and ethics reflection groups have emerged in an effort to provide the support sought by staff in these difficult circumstances. Outcomes of moral case deliberation, for example, relate to better collaboration with co-workers, as well as increased moral self-reflection and increased sensitivity and ability to recognize ethically relevant situations.^42,43^ Ethical skills are pivotal when dealing with ethically difficult situations. However, nurses facing the challenges of prioritization also highlight the need for boundaries.^32,34^ What is safe and competent nursing care? Where is the limit/threshold for safe and competent provision of care? These questions are difficult to answer, as each situation is unique and context-bound, diversified, and complex. However, we can start to attempt to answer them by identifying and describing what safe, competent nursing care is and reflecting on and discussing these issues.

### Matter of concern for next of kin

A consequence of limited resources is that next of kin often become family caregivers. The role of family caregiver influences both the caregiver’s relation to the patient and their life in general,^44^ often implying an experience of care burden, role conflicts, and a need for support.^45^ Research emphasizes the need to clarify both how to systematically involve and support family caregivers^32,44,46^ and what responsibilities the family caregiver role entails.^45^ Thus, it is necessary to find a balance between next of kin’s involvement as caregivers and the nurses’ responsibilities for providing safe, competent nursing care. Specifying what constitutes safe, competent nursing care will provide boundaries of involvement and thus support nurses in decision-making, as well as providing patients and their next of kin with an understanding of what to expect when in need of nursing care.

To summarize, for patients, nurses, and next of kin, it will be helpful to illuminate the content and boundaries of safe, competent nursing care in order to ensure that individual patients receive the care they need. However, setting boundaries for safe, competent nursing care also has consequences for fundamental values in nursing, as well as for how we might consider what is meant by a right to healthcare. These matters will be elaborated on in the next section.

## Fundamental values and the right to healthcare

When patients’ needs for nursing care are disregarded as a result of prioritization and rationing, fundamental nursing values and human rights are threatened.

According to the International Council of Nurses (ICN), respect for human rights, including cultural rights and the right to life and choice, dignity, and the right to be treated with respect, is inherent in nursing.^47^ Thus, fundamental values in nursing relate to both the human right to healthcare and moral values. Moral values are “*values of a distinctively moral nature in that they derive from the significant moral interests people have in upholding such things as human life, freedom and self-determination, welfare and well-being*.”(p.7)^36^ Ethics has to do with processes we use to ascribe fundamental values to human actions, behaviors, institutions, and character traits^48^ and, in turn, justify those ascriptions.^36^ Hence, to justify nurses’ conduct related to a minimum standard of safe, competent nursing care, fundamental values such as moral values have to be ensured, as does the right of humans to healthcare.

Human rights entail the right to a universal minimum standard of health and healthcare, including a minimum standard of nursing care.^49^ However, there are debates related to the interpretation and application of this right to healthcare, including how to define health, what minimum entitlements are encompassed in the right to healthcare, and which institutions are responsible for ensuring a right to such healthcare. These questions are also socioeconomic/political ones. What could be deemed a minimum acceptable standard of nursing care will look different in a well-funded Norwegian acute hospital versus an African rural hospital largely dependent on voluntary donations as a source of funding. Thus, while the upper limit of when missed nursing care, rationing, care left undone or unfinished threatens human rights may differ, perhaps the lower limit does not. The World Health Organization (WHO) uses the term people-centered care, which focuses on the health needs and expectations not only of individuals but also of families, communities, and society as a whole. People-centered care encompasses the clinical encounters and attention to the health of people in their communities, in addition to health policy and health services.^50^ Hence, when discussing the content of safe, competent nursing care, we must relate the minimum standard of care to the context in which it is provided, including the resource base in the country, as well as to the role of next of kin and cooperation between the different care providers.

However, ultimately only nurses and members of the nursing profession have the competence to set this minimum standard, as they have the knowledge and skills to set the lower limit of what constitutes safe, competent nursing care.

What is proposed here are key principles of a framework to articulate a minimum standard, as a point of departure for the nursing profession in each country to start developing context-sensitive minimum standards. These minimum standards should apply to each specific setting and take into consideration the society and legislation in which nursing care is provided. While the standards proposed here do have a common content (areas of concern and values to preserve), what is considered qualitatively satisfactory will vary depending on patients’ needs and context. We cannot operate with a fixed standard as a set of numbers, for example granting all patients in home care showers once a week. We must operate according to personal and situational needs. That is, some patients need to shower every day and some only once a week. In addition, we must operate according to national standards and what is reasonably affordable in a particular socioeconomic context.

In developed countries where healthcare is a tax-funded public service, healthcare such as nursing care is embedded in legislation.^16^ Hence, safe, competent nursing care is a patient right and is considered a part of the welfare state’s safety net. What this implies in concrete terms, however, is often unclear. In Norway, government documents highlight that prioritizations should not violate fundamental values^51^ and safe, competent nursing care is regarded as a fundamental value that cannot be compromised by limited resources.^52^ Nevertheless, rationing is highly evident in Norwegian healthcare services.^28,30–35^

## The Norwegian case—a point of departure

As an example of legislation in force setting boundaries for safe, competent nursing care, we use Norwegian legislation as a point of departure. Norwegian legislation emphasizes inhabitants’ legal right to necessary healthcare.^53^ Necessary healthcare includes provision of nursing care services, as the regulation aims to ensure that patients’ fundamental nursing care needs are met. In addition, the legislation gives further guidelines outlining what these fundamental needs encompass.^54^ Thus, it seems suitable to further examine the content of fundamental nursing care within the context of Norwegian legislation.

### Fundamental nursing and care services in Norwegian legislation

The statutory basis for nursing care services in Norway is the Municipal Health and Care Services Act of 2011,^53^ which states that all Norwegian municipalities have a duty to provide necessary health and care services to inhabitants who require it. The Act does not give patients the right to specific services. However, the aims, content, and tasks of the healthcare services are described in REG 2003-06-27 No. 792: *Regulation relating to quality in nursing and care services*.^54^ The regulation gives the municipalities the responsibility of developing procedures to ensure patients fundamental needs are met, which are defined as follows; (Table 1).

**Table 1.** Patients’ fundamental needs.

REG 2003-06-27 No. 792: Regulation relating to quality in nursing and care services: • Experience respect, predictability, and security in relation to service provision • Independence and control over one’s own life • Physiological needs such as adequate nutrition (food and drink), a varied and healthy diet, and a reasonable choice of food • Social needs such as the possibility for the company of other people, social contact, fellowship, and activity • Follow a normal life course and daily rhythm and avoid being bedridden when this is undesirable and unnecessary • The possibility for a peaceful and protected private life • Attention to personal hygiene and natural functions (toilet) • The possibility to take care of oneself • Dignity in the terminal phase of life in safe and peaceful surroundings • Necessary medical treatment, rehabilitation, nursing, and care adapted to the needs of the individual • Necessary dental treatment and attention to oral hygiene • Services especially adapted for people with dementia and other people who have problems expressing their needs • Appropriate help with meals and sufficient time and peace to eat • Appropriate help to dress and undress • The offer to participate in varied and appropriate activities

These are the fundamental needs that nurses are expected to meet vis-à-vis their patients. These are also the needs patients (and their relatives) can expect nurses to attend to when providing nursing care. Fundamental needs entail physiological, psychological, social, and spiritual needs, as well as emphasizing moral values such as respect, autonomy, integrity, and so on in provision of care. Thus, professional ethical conduct and skills play an important role in the fulfillment of these fundamental needs. Managerial factors, which include values such as predictability and security, also contribute, especially when fundamental needs refer to following a normal life course and daily rhythm. Table 2 summarizes aspects of fundamental nursing and care services according to Norwegian legislation (Table 2).

**Table 2.** Aspects of fundamental nursing and care services according to Norwegian legislation.

*1. Knowledge and clinical skills:* Nurses must have knowledge about basic principles of nursing and the clinical skills to make assessments and judge when nursing care is needed to meet fundamental physiological, psychological, social, and spiritual needs.
*2. Values, moral sensitivity, and skills:* Provision of fundamental nursing and care services must safeguard moral values, such as dignity, respect, security, autonomy, and empowerment. Safeguarding moral values requires nurses to possess the needed attitudes and skills to act in compliance with professional conduct, as well as having implications for managerial issues.
*3. Managerial capability and capacity:* To enable nurses to provide fundamental nursing and care, the service must be organized in such a way that nurses can fulfill the purpose of the service and meet patients’ needs in compliance with professional norms and moral values. This entails using a framework for safe staffing and skill-mix and implies having the resources to ensure that such frameworks and the findings from them can be implemented. That is, if the safe staffing framework indicates the need for an extra nurse on duty to provide adequate care, then it must be possible to access and employ the additional nurse.

Furthermore, Norwegian legislation also sets minimum standards called *the requirement of reliable and caring services*, a responsibility of all healthcare personnel to provide safe, competent, and attentive healthcare services, which we elaborate in the following section of this article.

### The requirement of reliable and caring services

In accordance with the Norwegian Health Personnel Act (LOV1999),^55^ all health personnel, including nurses, “shall conduct their work in accordance with the requirements of professional responsibility and diligent care” (§4), also called *the requirement of reliable and caring services.* That is, health personnel are obliged to provide clinically sound and caring services and are expected to conduct their work in accordance with their “qualifications, the nature of their work and the situation in general.” Hence, the extent to which professional conduct and judgment are expected depends on education, place of work (such as hospital, nursing home, or home-based care), and other elements influencing the work of the nurse—such as staffing level and skill-mix. Each professional healthcare worker has a personal responsibility to provide sound and caring services. However, institutions have the responsibility to organize and enable healthcare workers to perform their work according to legal rights and codes of professional conduct. Hence, there is an important connection between an individual nurse’s ability to provide sound and caring services and the context in which these services are provided. If nurses, or other professional healthcare workers, are not able to provide sound and caring services due to organizational constraints such as low staffing level or too large a workload, the organization is obliged to take measures to improve this situation. Thus, *the requirement of reliable and caring services* is a responsibility bestowed upon professionals and health service leaders throughout all levels in organizations providing healthcare services in Norway. *This means that we have to take into account contextual factors, including both where a service is provided and the situation surrounding the service provision, when setting a minimum standard.*

In Norway, *the requirement of reliable and caring services* sets the legal minimum standard for provision of healthcare and for all professional conduct. According to Norwegian law, professionals either provide sound and caring services or they do not.

*However, this legislative minimum standard must be filled with content according to each profession’s own guidelines and judgment. Three criteria are set for professional assessment of sound and caring services*:^56^

1. Provision of services must be qualitatively satisfactory and caring.
2. Provision of services must have sufficient scope.
3. Services must be provided in time

*The requirement of reliable and caring services*, then, refers to professional norms and guidelines describing best practices.^52^ That is, nursing care must be provided according to accepted and acknowledged practice within the nursing profession. However, this requirement is not a precise, fully articulated concept but rather serves as a point of departure when outlining and setting limits for a minimum standard of care.

Thus, it is for us in the nursing profession to decide how great a deviation from the professional norms and guidelines of best practice we can accept before our care does not meet the *requirement of reliable and caring services.* The question, in other words, is when does the provision of nursing care deviate from the norms to such an extent that nurses will proclaim the service unsound and/or uncaring? Or, put another way, when can the norms be considered broken such that clinically sound and caring services are no longer provided? When are services not qualitatively satisfactory? When does the provision of services not have sufficient scope and when are they not provided in time? These are pivotal questions we need to answer to be able to say what safe and competent nursing care is and not.

## Safe, competent nursing care—a suggested framework

Nursing theorists and researchers have long sought to identify what fundamental nursing care entails, starting with Nightingale^13^ and continuing with, among others, Henderson^14^ and the 14 basic components of nursing care and, more recently, Kitson^15^ and the fundamentals of care. Moreover, researchers have developed core elements to help set minimum standards.^57^ However, there is a lack of consensus about how to define the fundamentals of nursing care, although the need to agree on some kind of definition has been acknowledged.^58^ The description of fundamental needs and care in Norwegian legislation and the requirement to provide clinically sound and caring services seem, however, to capture important aspects of what safe and competent nursing care might encompass. There is a minimum standard (lower limit) based on human rights and patient needs that must be met if effective, safe, competent nursing is taking place. This includes humane, respectful, comfort care, hydration, basic nutrition, and so forth. However, beyond that, patient expectations, not to mention sociopolitical, economic, and cultural circumstances, will vary, and the upper limit of safe, competent, human care is much less clear and less easy to determine and ultimately merges into comprehensive care. Hence, we suggest a framework of safe, competent nursing care that includes patients’ fundamental needs, values to preserve in provision of fundamental care, and a minimum standard of care adjusted according to setting and context and meeting the requirement of reliable and caring services. In table 3 below we suggest aspects to consider for developing a minimum standard of safe competent nursing care.

**Table 3.** Aspects for developing a minimum standard of safe, competent nursing care.

Aspects to consider when developing a minimum standard of safe, competent nursing care: *1. Provision of care according to patients’ fundamental physiological, psychological, social, and spiritual needs.* *2. Attention to fundamental needs such that moral values are safeguarded and in line with nurses’ professional code of ethics.* *3. Determination of the lower limit of fundamental care must meet the requirement of reliable and caring services according to setting and socioeconomic context in the relevant country.*

Hence, safe, competent nursing care refers to something more than just life-saving treatment but less than optimal comprehensive care. There is a difference between maximum standards of care and minimum standards that we can agree are sufficiently clinically sound and caring to meet the requirement established by law. To set this minimum standard, more details about the particular context in which care is being provided are necessary. We suggest four questions nurses can use to evaluate whether the care they provide meets the requirement of reliable and caring services^52^ or not (See Table 4).

**Table 4.** Evaluation of safe and competent services.

Four questions to evaluate whether nursing care is provided in a safe and competent manner in your setting, thus meeting the criteria of reliable and caring services: *1. Are all the patients’ fundamental needs met in a qualitatively satisfactory way, that is, in accordance with professional norms and values?* *2. Is the scope of service provision sufficient to meet all patients’ fundamental needs adequately, that is, according to professional norms and values?* *3. Are all the patients’ fundamental needs met in time?* *4. Are the acts of nursing caring and empathic?*

If the answer to these questions is yes, safe, competent nursing care is being provided and the minimum standard of nursing care can be considered met. If the answer is no, the minimum standard is not met.

Determining what constitutes safe and competent nursing care is difficult, but important. Setting a lower limit for the standard of care, below which care is determined to be inadequate and unacceptable, might empower nurses, patients, and next of kin in clinical healthcare settings, as well as prevent negative outcomes. It will, however, probably require organizational and managerial changes. Standards can function as normative thresholds for health services and ensure safe and competent nursing care. The challenge is, of course, determining who has the legitimacy to develop and decide the content of these standards and who shall be obliged to meet and enforce them. Should there be sanctions if standards are broken or not upheld?

## Conclusion

In this article, we suggest that a minimum standard of safe and competent nursing encompasses basic principles of nursing care, moral values, and principles of professional conduct in compliance with the nursing code of ethics. Norwegian legislation is used as a point of departure, as it is compatible with nursing theory, fundamental nursing values, and the human right to healthcare. However, perceptions and understandings of the dimensions of safe, competent nursing care need to be adjusted to account for setting, context, and country to ensure a reasonable and sustainable description of safe, competent nursing care that also takes into account the needs of the particular patient. If we are to consider nursing care to meet the minimum standard and the *requirement of reliable and caring services*, the answer must be “yes” to the four pivotal questions above.
